# Findings of a Multidisciplinary Assessment of Children Referred for Possible Neurodevelopmental Disorders: Insights from a Retrospective Chart Review Study

**DOI:** 10.3390/bs12120509

**Published:** 2022-12-14

**Authors:** Shuliweeh Alenezi, Aqeel Alkhiri, Weaam Hassanin, Amani AlHarbi, Munirah Al Assaf, Norah Alzunaydi, Salma Alsharif, Mohammad Alhaidar, Abdulaziz Alnujide, Fatimah Alkathiri, Abdulaziz Alyousef, Razan Albassam, Hadeel Alkhamees, Ahmed S. Alyahya

**Affiliations:** 1 Department of Psychiatry, College of Medicine, King Saud University, Riyadh 11451, Saudi Arabia; 2 Department of Psychiatry, King Saud University Medical City, King Saud University, Riyadh 12372, Saudi Arabia; 3 SABIC Psychological Health Research and Applications Chair (SPHRAC), Department of Psychiatry, College of Medicine, King Saud University, Riyadh 11451, Saudi Arabia; 4 Department of Mental Health, Al Qunfudah General Hospital, Al Qunfudah 28821, Saudi Arabia; 5 Prince Mohammed Bin Salman Center for Autism and Developmental Disorders, Prince Sultan Military Medical City, Riyadh 12426, Saudi Arabia; 6 Department of Psychiatry, Eradah Complex for Mental Health, Riyadh 12571, Saudi Arabia

**Keywords:** autism spectrum disorder, multidisciplinary team assessment, developmental disorders, Saudi Arabia

## Abstract

Children with ASD have a wide spectrum of functional deficits in multiple neurodevelopmental domains. A multidisciplinary team assessment (MDT) is required to assess those deficits to help construct a multimodal intervention plan. This is a retrospective chart review of the assessment for children who were referred for an assessment of potential neurodevelopmental disorders. We reviewed 221 participants’ charts from January 2019 to January 2020. The mean age of the children was 7.95 ± 3.69, while the mean age of the fathers and mothers was 37.31 ± 8.57 and 31.95 ± 6.93, respectively. Consanguinity was as high as 37.9% for the referred children with developmental delay who were first-degree related, and 13.2% of the parents were second-degree relatives. Approximately 26.6% of children had a family history of mental illness in first-degree relatives. ASD was the most commonly reported diagnosis post-assessment, and ADHD was the most common reported comorbidity at 64.3% and 88.5%, respectively. The MDT findings showed that 58% of children required moderate or higher assistance with toileting, 79.2% were unable to answer yes/no questions, and 86.8% were unable to understand “wh” questions. Only 26% of the nonverbal children had average IQ testing results, and 31% of verbal children did. In conclusion, the mean age of the children when assessed was above that recommended for early screening and intervention. An increased paternal and maternal age was noticeable. Consanguinity and a family history of mental disorders in first-degree relatives were high, attesting to a possible genetic risk.

## 1. Introduction

Neurodevelopmental disorders are a group of disabilities that are primarily associated with functional disturbances of the brain and neurological system. They encompass a wide range of disorders, including autism spectrum disorder (ASD), attention deficit and hyperactivity disorder (ADHD), intellectual disability (ID), and motor, communication, learning, and language disorders [1]. Estimates of the prevalence of developmental disability differ among various populations and settings according to different methodological approaches. Data from a study in the United States estimated a prevalence of 15.04% for any developmental disability in children aged 3 to 17 years old [2]. Among them, ADHD was one of the most prevalent disorders. In 2015, a meta-analysis of 175 studies reported an overall pooled estimate of 7.2% [3]. The Saudi National Mental Health Survey (SNMHS) estimated that the lifetime prevalence and 12 month prevalence of ADHD in Saudi Arabia are 8% and 3.2%, respectively [4,5]. The prevalence of ASD worldwide was estimated to be approximately 52 million cases according to the Global Burden of Disease study in 2010, equivalent to 1 in 132 individuals [6]. In Saudi Arabia, data on the national prevalence of ASDs are scarce. A few studies have cited a prevalence of 18 per 10,000 and one in 167 individuals [7,8].

People with neurodevelopmental disorders experience difficulties in multiple domains, including cognition, learning, language and speech, and sensory, motor, and behavioral problems, thus prompting the need for comprehensive multidisciplinary assessments and interventions [9]. The evidence is abundant for the benefits of early diagnosis and intervention for neurodevelopmental disorders, leading to better functional outcomes [10]. This field faces multiple challenges in the Arab region, particularly in Saudi Arabia, which can be attributed to factors such as the scarcity and limitations of epidemiological studies, the shortage of trained professionals, and late access to diagnosis and early interventions [8,11].

Cognition is one of the domains commonly affected in people with neurodevelopmental disorders; it refers to the mental processing of information, including attention, memory, learning, decision making, reasoning, and problem solving. There is a high degree of heterogeneity in the underlying cognitive impairment in these disorders; furthermore, the high rates of comorbidity between them complicate the degree and extent of these problems, imposing even more challenges for research and clinical practice [12]. People with autism experience specific deficits in processing social and emotional information, which are common diagnostic features of ASD [13]. Furthermore, there is considerable comorbidity between ASD and ID. Some authors reported that 31% of children with ASD have comorbid ID [14]. In ADHD, the cognitive deficits frequently reported include executive dysfunction and information processing speed, and many individuals with ASD and ADHD scored lower in working memory and processing speed in intelligence testing [15]. The assessment of a child’s intellectual ability is considered a standard part of the evaluation of people with neurodevelopmental disorders worldwide [16], and even though it comes with its challenges, given the unique cognitive profile of an individual with these disorders, intelligence testing scores can play a role in designing a personalized care plan and placement into clinical and educational settings [17].

In terms of sensory functioning, children with neurodevelopmental disorders respond differently to sensory experiences than typically developing peers [18]. There is abundant evidence of sensory dysfunction in people with ASD, reflected by including sensory aspects in the latest Diagnostic and Statistical Manual of Mental Disorders (DSM-5) [1]. Many people with ASD experience sensory processing dysfunction (SPD), as it is challenging to receive, organize, and interpret sensory stimuli from various sensory systems [19]. These experiences may appear as hyper, hyperreactivity, or seeking a particular sensory stimulus. It is estimated that as high as 90% of people with ASD have sensory abnormalities [20]. A study among 64 children diagnosed with ASD in Riyadh, Saudi Arabia, found that 84% had significant sensory dysfunctions [21]. In ADHD, several reports suggest the presence of abnormal sensory processing in multiple domains when comparing ADHD with control groups [22]. A study comparing the sensory profiles of children with ADHD or ASD and typically developing children found that ADHD and ASD were associated with more sensory processing deficits than children without disabilities [23]. The presence of sensory dysfunctions in children with neurodevelopmental disorders implicates their daily activities, interactions, and learning experiences [24]. These findings reflect the vitality of an assessment for early detection, paving the way for evidence-supported early intervention programs [25]. Even though SPD is largely seen in people with different NDDs, a specific pattern for each disorder has not established yet. In addition, people sharing the same condition also showed different sensory processing patterns. This might be related to the different sensory thresholds and coping strategies in each individual. Moreover, additional factors, such as age, gender, and being medicated, play a role in this variation of sensory processing within people with the same condition [26,27,28,29]. 

Language and speech are among the domains that are frequently affected in children with neurodevelopmental disorders. Communication delays are often among the first issues that indicate ASD [30]. However, there is high heterogeneity in the ability of children with ASD to understand and use language [31], with some children experiencing severe language impairment, while other children’s language abilities are comparable to typically developing children [32]. Multiple levels of speech and communication can be affected, including nonverbal (e.g., facial expressions, gestures, and eye contact), paralinguistic (e.g., prosody and intonation), and linguistic level (e.g., language and speech). Deficits in the social use of speech and language are particularly prominent in people with ASD. Furthermore, in a systematic review of 21 studies, children with ADHD were found to have poorer language function than controls in multiple language domains, including overall language performance, expressive, receptive, and pragmatic languages [33]. Language and speech problems can result in several adverse outcomes, such as behavioral difficulties, poor adaptive skills, poor social skills, and poor academic function [34,35]. However, the earlier a child acquires speech and language skills, the better the outcomes are in adaptive and social functioning [36]. Furthermore, some evidence suggests a lower efficacy of such intervention when applied after five years, which emphasizes the importance of earlier detection and management [37].

Children with neurodevelopmental disorders present with complex issues affecting multiple aspects of their development. This asserts the role of multidisciplinary team evaluation in addressing these issues. This study aimed to explore the findings of a multidisciplinary team assessment of children referred for possible neurodevelopmental disorders.

## 2. Methods

### 2.1. Study Design and Setting

This was a retrospective chart review study of children who were referred for assessment of potential neurodevelopmental disorders to Prince Mohammad Bin Salman Center for Autism and Developmental Disorders (PMBSC-ADD) at Prince Sultan Military Medical City (PSMMC), Riyadh, Saudi Arabia. The PMBSC-ADD is a specialized center that provides diagnostic services and interventions for patients with autism and developmental disorders. The center receives referrals from different services within the Ministry of Defense’s health care system.

The Institutional Review Board approved the study at PSMMC on March 2020 (Ref. No.: 20/0626/IRB). The study reviewed the charts of children referred to the center between January 2019 and January 2020.

The diagnostic evaluation was performed by a multidisciplinary team of professionals, including physicians (child and adolescent psychiatrists), psychologists, speech and language pathologists, occupational therapists, and behavioral therapists. The diagnosis of neurodevelopmental disorders was made clinically following an extensive assessment procedure, including clinical interviews according to DSM-5 criteria and obtaining developmental and psychosocial information from parents, guardians, or other caregivers.

For children referred for occupational therapy assessment, sensory processing characteristics were evaluated using the Sensory Profile 2 (SP-2) [38]. Daily living skills were evaluated using the Functional Independence Measure (WeeFIM), a validated tool used to determine children’s level of independence in DLS [39]. The speech and language pathologist conducted a clinical examination of the referred children for language and communication assessment. Their assessment included inquiry concerning hearing assessments, feeding issues, pre-communication, nonverbal communication skills, and expressive and receptive language abilities. Licensed psychologists performed the psychological and cognitive assessments. Adaptive skills were assessed with the Vinland Adaptive Behavior, second edition (VABS-II), a semi-structured interview for caregivers to evaluate a child’s adaptive skills in four areas: communication, daily living, socialization, and motor skills [40]. The intelligence quotient (IQ) was assessed using the Stanford Binet Scale—Fifth Edition (SB-5) for children with verbal abilities [41], and the Leiter International Performance Scale-Revised (Leiter-R) for nonverbal children [42].

### 2.2. Procedure

The sociodemographic information, clinical data, and results of the standardized assessment tests and scale of the child were collected from electronic medical records using a data collection form designed specifically for this study. The members of the research team, who were part of the multidisciplinary team conducting the assessment and are familiar with the terminology around NDD, were responsible for reviewing and collecting the charts. Data were stored on a secure computer at the research center and used only for research purposes.

## 3. Statistical Analysis Plan

Data collection and preparation were conducted using Microsoft Excel. The data analysis was performed using IBM SPSS version 28. The descriptive statistics are presented as numbers and percentages for the categorical variables, while the minimum, maximum, mean, and standard deviation were used for the numeric variables.

## 4. Results

### 4.1. Characteristics of the Study Sample

Table 1 shows, that a total of 221 cases were included in this study. A total of 96.8% of the sample were Saudis; 74.2% of the study sample were males, and 25.8% of the sample were females. Of the study sample, 7.8% had less than 5000 Saudi Riyal (SAR) of family monthly net income, 29.3% of the sample had a 5000 to 10,000 SAR of family monthly net income, 36.5% of the sample had 10,000 to 15,000 SAR of family monthly net income, and 26.3% of the sample had a family monthly net income more than 15,000 SAR.

A total of 37.9% of the parents in the study sample were first-degree related, 13.2% were second-degree related, while 48.9% were not related. In addition, 26.6% had a family history of mental illness in first-degree relatives, 52.7% had previous psychiatric medication use, and 24.3% reported current psychiatric medication use.

Table 2 describes the age of children referred for assessment and their parents. The mean age of the children was 7.95 ± 3.69. The mean age of the fathers when their child was born was 37.31 ± 8.57, while the mean age of the mothers when their child was born was 31.95 ± 6.93.

### 4.2. Diagnosis

Table 3 shows both the primary and secondary diagnosis based on a clinical evaluation. Our sample showed that 64.3% of the children were primarily diagnosed with ASD, 9.8% with ADHD, while only 0.4% of the children were primarily diagnosed with ODD. Moreover, 88.5% of the patients were secondarily diagnosed with ADHD.

### 4.3. Occupational Therapy Assessment

#### 4.3.1. Sensory Profile

Table 4 and Table 5 show the OT assessment findings for the children and toddlers. A total of 121 children had an assessment using the children’s sensory profile. For the children’s sensory profile, the mean seeking quadrant score was 38.29 ± 10.90, the mean avoiding quadrant score was 40.46 ± 11.01, the mean sensitivity quadrant score was 39.79 ± 9.48, and the mean registration/bystander quadrant was 38.50 ± 8.93. The mean auditory processing score was 18.29 ± 5.29, the mean visual processing score was 11.86 ± 3.34, the mean touch processing score was 17.26 ± 5.73, the mean movement processing score was 15.24 ± 4.36, the mean body position processing score was 11.33 ± 3.68, and the mean oral sensory processing score was 19.16 ± 7.55.

The mean conduct associated with the sensory processing score was 21.73 ± 6.36, the mean social–emotional responses associated with sensory processing was 28.28 ± 8.16, and the mean attentional responses associated with the sensory processing score was 23.21 ± 7.47.

A total of 19 children had an assessment using the toddler sensory profile. For the toddler sensory profile, the mean seeking/seeker quadrant score was 21.53 ± 6.34, the mean avoiding/avoider quadrant score was 19.26 ± 5.81, and the mean sensitivity/sensor quadrant score was 23.89 ± 8.04. The mean auditory processing score was 18.00 ± 5.04, the mean visual processing score was 12.32 ± 3.53, and the mean touch processing score was 9.32 ± 3.48. The mean movement processing score was 13.21± 3.69, the mean oral sensory processing score was 10.68 ± 4.31, and the behavioral responses associated with sensory processing was 13.37 ± 5.64.

#### 4.3.2. WeeFIM Levels

The results of the children’s assessment using the WeeFIM tool for eating, grooming, bathing, dressing—upper body, dressing—lower body, and toileting are presented in the table above. The percentage of the study sample that needed total assistance was 2.3% for eating, 14.5% for grooming, 18.3% for bathing, 3.8% for dressing—upper body, 4.6% for dressing—lower body, and 49.2% for toileting. (Table 6).

### 4.4. Speech and Language Assessment Findings

#### 4.4.1. Pre-Communication Assessment

Table 7 shows the findings for the pre-communication skills. The most frequent categories were as follows: 45.4% of eye contact was fair, 50.7% of verbal imitation was poor, 42.7% of nonverbal imitation was poor, and 42.0% of social interaction was poor. While for the nonverbal communication skills, the most frequent categories were as follows: 51.3% of pointing was poor, 46.0% of gestures was poor, 45.9% of facial expression was good, 55.7% of the receptive language level was a severe delay, 63.4% of the expressive language level was a severe delay.

#### 4.4.2. Speech and Language Therapy Communication Assessments Findings

Table 8 describes the findings of the SLP assessment. The mean SLP sessions since the SLP assessment was 5.81 ± 7.91 SD, while the mean chronological age was 65.49 months ± 33.93 SD. A total of 24.4% of the children had feeding concerns, while 5.6% of them had hearing loss.

A total of 79.2% of the children could not understand yes/any questions, while 86.8% of the children could not understand “wh” questions, and 32.1% of the children could not respond to their name. Moreover, 19.6% of the assessed children had echolalia.

### 4.5. Psychological Assessment

Table 9 and Table 10 show the findings of the psychological assessment. Our sample showed that 80% of the children had low communication, 57% of the children had low daily living skills, 72% of the children had low socialization, 58% of the children had low motor skills, and 78% of the children had a low adaptive behavior composite. Only a small percentage of the study sample was assessed for IQ using either the IQ Leiter test or the SB5 test, and 26% of the children had an average delay in the IQ Leiter test, while 25% of the children had an average score on the SB5 test. 

## 5. Discussion

In this study, we explored the findings of a multidisciplinary assessment of 221 children referred for the assessment of possible neurodevelopmental disorders. Two-thirds of the referred children were diagnosed with ASD, followed by ADHD in 10%, and 8.5% were not diagnosed with neurodevelopmental disorders. When assessed for comorbidities, 46 children had comorbid ADHD in addition to the primary diagnosis. Children with an NDD show high comorbidities, especially with other NDDs. Some studies found that 40 to 80% of individuals with ASD also have ADHD [43]. In this study, the male-to-female ratio in our sample was 2.8 boys to one girl. This result is consistent with the prevalence ratio in the literature for neurodevelopmental disorders, which varies between two and four males to one female [44]. Similar to these findings, two Saudi Arabia studies reported a ratio of 2.7:1 and 3.5:1 boys to girls, respectively, among children diagnosed with ASD [11,45].

Parental consanguinity is a prevalent phenomenon in Saudi Arabia and the Middle East, with previous studies from SA reporting a prevalence between 28.5% and 39% of the consanguineous relationship between the parents of children with ASD [11,45,46]. In the current study, the consanguinity rate was 51.1%, exceeding that of the studies mentioned above. This variation could partly be attributed to our sample’s heterogenicity, as there were multiple neurodevelopmental disorders in addition to ASD, compared to sample populations of ASD only in the previous studies. This gap in the relationship between consanguinity and neurodevelopmental disorder among the Saudi population is worth highlighting, and future research on this issue is recommended. Another finding of this study is the history of mental illness in 26.6% of first-degree relatives. A cohort study in Sweden found an increased risk of autism when there is a family history of multiple mental and neurological disorders [47]. These results also align with multiple studies that found an increased risk of ASD in children with a history of mental illness in first-degree relatives [48,49]. This study found that approximately half of the patients with an NDD (52.7%) had a history of psychiatric medication use, which is higher than a previous study from Saudi Arabia, which only found that 39.08% of children with ASD were on psychiatric medications [50]; a range between 2.7% and 80% has been reported in the literature [51]. This increase in psychiatric medication use could be partly related to the limited access to nonpharmacological services for children with NDDs. 

Social communication and language delay are among the most frequently reported concerns in children with NDD. In the current study, most children were found to have low communication and socialization skills and language delays. Furthermore, most children who went through speech and language assessment demonstrated poor levels of pre-communication and nonverbal communication skills. It has been estimated that approximately 30% of children with ASD have minimal verbal abilities and varying degrees of severity in communication and language impairments [52]. Multiple studies reported social communication impairments and language delay as the most common reason for the referral of children with ASD for assessment [11,46,53]. 

Cognitive abilities in individuals with NDDs may be a key indicator of long-term outcomes and has an essential impact on developing treatment goals. Only 43 children in this study went through an IQ assessment. Most of those children scored lower than average, with 60% and 44% receiving scores suggestive of ID on Leiter and Stanford-Binet intelligence scales, respectively. Recent studies reported a 30%–50% rate of intellectual disability among children with ASD [14,54]. The higher ID rates in our sample may be because only a small number of children had an IQ assessment. The heterogenicity in intellectual functions within the NDD population leads to multiple challenges when considering intervention plans. Reports often indicate that the presence of an intellectual disability is associated with poor social communication and language skills, motor abilities, and slow improvement in the acquisition of daily skills [55,56]. 

The current results found that children with NDD scored high in children and toddler sensory profiles. The most common sensory profiles included avoiding, sensitivity, registration, and seeking domains. These results are similar to those of Narizisi et al. [52], who found that children diagnosed with autism have a higher prevalence of sensory alteration than typically developing peers in Italy. Another study among Saudi Arabian children, by Al-Heizan et al., reported similar results, with a higher prevalence of sensory dysfunction in children with ASD [21]. Other studies have pointed to the effect of such sensory difficulties on social communication [57], more severe restricted and repetitive behaviors [58], feeding [59], and sleep problems [60] in children with NDDs. The results of our current study indicate increased rates of motor difficulties in most children with NDDs, with 31% and 58% scoring below average and low in the motor skills domain, respectively. These results are consistent with previous studies reporting poorer motor skills among children with developmental disabilities [61,62]. The increase in both sensory and motor difficulties has been associated with reduced daily living skills performance [63]. These findings show the importance of sensory and motor assessments of children with NDDs and implementing standard treatment programs with occupational and physical therapy.

In this study, most children with neurodevelopmental disorders had low adaptive behavior composites and scored low in all domains of adaptive behavior skills measured by VABS-II. These results are similar to the reported profiles of children with neurodevelopmental disorders in the literature [64,65]. We also explored the daily living skills among children with NDDs. Our results show that most children with NDDs (88%) had low or below-average daily living skills, similar to another study that reported a lack of self-care skills among 82.9% of school-aged children with ASD [66]. In this sample, most of the children required assistance with most domains of daily living skills (DLS), with bathing, upper body dressing, and toileting requiring the highest level of assistance, followed by grooming, lower body dressing, and eating. These findings are consistent with a Turkish study that assessed the DLS of children with ASD using the WeeFIM II scale [67]. They reported that their participants needed assistance in most DLS. However, there was a difference in the domains that needed help compared to our sample, with bathing and grooming requiring the highest level of assistance, while upper and lower body dressing required lower levels of assistance. These differences could be due to the inclusion of children with other NDDs and younger ages compared to a sample consisting mainly of children with ASD who were above thirteen years old in the Turkish study.

These findings reflect the need for a multidisciplinary team assessment to address the range of difficulties encountered by children with NDDs. Although the support for better outcomes with an earlier intervention is mixed [68], multiple studies indicated more robust and beneficial results with early recognition and intervention for people with NDDs [10,25]. Our results show that the average age of the referred children was 7.95 years. This is inconsistent with previous results that showed that the average age of an ASD diagnosis was between two and three years of age, reported by previous Saudi studies [11,45]. Similar to our findings, a previous Australian study, by Bent et al., reported a later age of diagnosis with an average age of 4 years, and commonly diagnosed at 6 years of age among their sample of children with ASD [69]. Several factors could contribute to the later age of diagnosis, including a less severe symptomatology, which may not have given rise to early concerns for parents. Another factor to consider is that social communication, attentional, and academic difficulties are indicative of NDD diagnoses and may not have been present until children began to attend kindergartens or preschools. There have been increasing efforts to address these gaps in delayed diagnosis and referrals for assessment and intervention in Saudi Arabia, with the national policy for screening for autism being approved in 2021 [70]. More recently, a consensus statement by Saudi experts in the field highlighted the importance of both early multidisciplinary team assessment and early interventions for children with autism in efforts to bridge these gaps [71].

## 6. Conclusions

When evaluated, the average age of the children was older than what is advised for early screening and intervention. The maternal and paternal ages were higher in our sample. Significant rates of consanguinity and a history of mental diseases were reported, indicating a potential hereditary risk. In our sample, a below average IQ or less was reported in a higher percentage of children referred due to the fact of a neurodevelopmental delay.

## 7. Limitation and Future Direction

Several limitations were addressed before conducting the study, such as inconsistent or inaccurate coding of chart information, by training data collectors and piloting to assess rater and inter-rater reliability. However, many other limitations of the chart review process might have influenced the current findings, such as missing charts and a lack of blinding to the study purpose. In addition, one of the limitations was that the speech and language assessment was an informal and non-standardized test for Arabic speakers, which might have affected the consistency of the result. Thus, developing a standardized speech and language assessment for Arabic speakers will improve the consistency and reliability of the results. Furthermore, neuroimaging and neurophysiological studies were not part of the current study, and including them in future investigations could help in providing a clearer picture and an opportunity to compare results with the existing literature. Studies on possible strategies to improve earlier screening and referral for multidisciplinary team assessments and interventions are needed.

## 8. Recommendation

The early screening and referral of children with suspected neurodevelopmental disorders to MDT assessment are needed to discover functional deficits in the domains of language, cognition, and ADLs. Referral for genetic testing is advisable, given the higher consanguinity in the Saudi population.

## Figures and Tables

**Table 1:** Sociodemographic data of participants.

		N	%
Nationality	Saudi	214	96.8%
	Non-Saudi	7	3.2%
Gender	Male	164	74.2%
	Female	57	25.8%
Father’s level of education	Illiterate	5	2.8%
	Primary school	8	4.4%
	Intermediate school	32	17.7%
	High school	56	30.9%
	Diploma	10	5.5%
	Bachelor	51	28.2%
	Masters	19	10.5%
Mother’s level of education	Illiterate	3	1.6%
	Primary school	12	6.5%
	Intermediate school	23	12.5%
	High school	52	28.3%
	Diploma	11	6.0%
	Bachelor	72	39.1%
	Masters	11	6.0%
Family monthly net income	Less than SAR 5000	13	7.8%
	SAR 5000–10,000	49	29.3%
	SAR 10,000–15,000	61	36.5%
	More than SAR 15,000	44	26.3%
Parental consanguinity: are parents related?	First degree	72	37.9%
	Second degree	25	13.2%
	Not related	93	48.9%
Family history of mental illness in first-degree relatives?	Yes	21	26.6%
	No	58	73.4%
Previous psychiatric medication use?	Yes	48	52.7%
	No	43	47.3%
Current psychiatric medication?	Yes	52	24.3%
	No	162	75.7%

**Table 2:** Age of affected children and age of parents.

	N	Minimum	Maximum	Mean	SD
Age	220	1	18	7.95	3.69
Number of sisters	196	0	9	1.49	1.45
Number of brothers	196	0	9	1.63	1.55
Patient’s order to other siblings	196	0	11	3.08	2.21
Father’s age when the child was born	178	22.0	85.0	37.31	8.67
Mother’s age when the child was born	175	18.0	60.0	31.95	6.93

**Table 3:** Diagnosis after diagnostic assessment.

Primary Diagnosis	N	%
ADHD	22	9.8%
ASD	144	64.3%
GDD	14	6.3%
ID	5	2.2%
Language disorder	15	6.7%
No diagnosis	19	8.5%
ODD	1	0.4%
SCD	4	1.8%
Secondary Diagnosis		
ASD	1	1.9%
ADHD	46	88.5%
Language disorder: oppositional defiant disorder	1	1.9%
Language disorder	3	5.8%
ODD	1	1.9%

**Table 4:** Sensory profile results for the children.

	N	Minimum	Maximum	Mean	SD
Seeking/seeker quadrant score	121	20	77	38.29	10.90
Avoiding/avoider quadrant score	121	17	67	40.46	11.01
Sensitivity/sensor quadrant Score	121	19	67	39.79	9.48
Registration/bystander quadrant	121	21	69	38.50	8.93
Auditory processing	121	8	32	18.29	5.29
Visual processing	121	6	23	11.86	3.34
Touch processing	121	8	39	17.26	5.73
Movement processing	121	7	30	15.24	4.36
Body position processing	120	7	26	11.33	3.68
Oral sensory processing	121	10	55	19.16	7.55
Conduct associated with sensory processing	120	9	36	21.73	6.36
Social emotional responses associated with sensory processing	120	12	56	28.28	8.16
Attentional responses associated with sensory processing	117	11	48	23.21	7.47

**Table 5:** Toddler sensory profile.

	N	Minimum	Maximum	Mean	SD
Seeking/seeker quadrant score	19	8	32	21.53	6.34
Avoiding/avoider quadrant score	19	12	35	19.26	5.81
Sensitivity/sensor quadrant score	19	13	41	23.89	8.04
Registration/bystander quadrant	19	9	30	21.42	5.74
General processing	19	11	40	23.58	7.97
Auditory processing	19	8	25	18.00	5.04
Visual processing	19	7	19	12.32	3.53
Touch processing	19	6	19	9.32	3.48
Movement processing	19	7	20	13.21	3.69
Oral sensory processing	19	7	24	10.68	4.31
Behavioral responses associated with sensory processing	19	6	28	13.37	5.64

**Table 6:** WeeFIM results.

		N	%
Eating	1: Total assistance	3	2.3%
	2: Maximal assistance	18	13.7%
	3: Moderate assistance	29	22.1%
	4: Minimal assistance	15	11.5%
	5: Supervision	23	17.6%
	6: Modified independence	11	8.4%
	7: Complete independence	32	24.4%
Grooming	1: Total assistance	19	14.5%
	2: Maximal assistance	21	16.0%
	3: Moderate assistance	27	20.6%
	4: Minimal assistance	22	16.8%
	5: Supervision	18	13.7%
	6: Modified independence	6	4.6%
	7: Complete independence	18	13.7%
Bathing	1: Total assistance	24	18.3%
	2: Maximal assistance	35	26.7%
	3: Moderate assistance	32	24.4%
	4: Minimal assistance	19	14.5%
	5: Supervision	13	9.9%
	6: Modified independence	2	1.5%
	7: Complete independence	6	4.6%
Dressing—upper body	1: Total assistance	5	3.8%
	2: Maximal assistance	26	19.8%
	3: Moderate assistance	35	26.7%
	4: Minimal assistance	27	20.6%
	5: Supervision	12	9.2%
	6: Modified independence	4	3.1%
	7: Complete independence	22	16.8%
Dressing—lower body	1: Total assistance	6	4.6%
	2: Maximal assistance	25	19.1%
	3: Moderate assistance	33	25.2%
	4: Minimal assistance	22	16.8%
	5: Supervision	16	12.2%
	6: Modified independence	6	4.6%
	7: Complete independence	23	17.6%
Toileting	1: Total assistance	64	49.2%
	2: Maximal assistance	4	3.1%
	3: Moderate assistance	8	6.2%
	4: Minimal assistance	15	11.5%
	5: Supervision	5	3.8%
	6: Modified independence	3	2.3%
	7: Complete independence	31	23.8%

**Table 7:** Pre-communication and nonverbal communication skills.

Pre-Communication Skills		N	%
Eye contact	Poor	56	36.8%
	Fair	69	45.4%
	Good	27	17.8%
Verbal imitation	Poor	75	50.7%
	Fair	28	18.9%
	Good	45	30.4%
Nonverbal imitation	Poor	64	42.7%
	Fair	28	18.7%
	Good	58	38.7%
Social interaction	Poor	50	42.0%
	Fair	36	30.3%
	Good	33	27.7%
Nonverbal communication skills			
Pointing	Poor	77	51.3%
	Fair	24	16.0%
	Good	49	32.7%
Gestures	Poor	69	46.0%
	Fair	23	15.3%
	Good	58	38.7%
Facial expression	Poor	46	31.1%
	Fair	34	23.0%
	Good	68	45.9%
Receptive language level	Mild delay	19	12.8%
	Moderate delay	47	31.5%
	Severe delay	83	55.7%
Expressive language level	Mild delay	11	7.2%
	Moderate delay	45	29.4%
	Severe delay	97	63.4%

**Table 8:** SLP assessment.

		Mean	SD
Chronological age in months		69.49	33.93
		**N**	**%**
Feeding concern	Yes	39	24.4%
	No	121	75.6%
Hearing loss	Yes	9	5.6%
	No	151	94.4%
		**N**	**%**
Understand “yes/no” questions	Yes	33	20.8%
	No	126	79.2%
Understand “wh” questions	Yes	21	13.2%
	No	138	86.8%
Responding to name	Yes	67	42.1%
	No	51	32.1%
	Sometimes	41	25.9%
Echolalia	Yes	31	19.6%
	No	127	80.4%

**Table 9:** Psychological assessment.

		N	%
Communication	Average	4	4%
	Below average	16	16%
	Low	79	80%
Daily living skills	High	1	1%
	Average	12	12%
	Below average	30	30%
	Low	58	57%
Socialization	Average	6	6%
	Below average	21	21%
	Low	71	72%
Motor skills	Average	8	11%
	Below average	23	31%
	Low	43	58%
Adaptive behavior composite	Average	3	3%
	Below average	18	19%
	Low	76	78%

**Table 10:** IQ test result.

		N	%
IQ Leiter	Average	7	26%
	Below average	2	7%
	Low	2	7%
	Mild delay	5	19%
	Moderate delay	6	22%
	Severe delay	5	19%
SB5	High average	1	6%
	Average	4	25%
	Low average	2	13%
	Borderline impaired or delayed	2	13%
	Mildly impaired or delayed	5	31%
	Moderately impaired or delayed	2	13%

## Data Availability

All of the data for this study will be made available upon reasonable request.
